# Fluorodeoxyuridine Improves *Caenorhabditis elegans* Proteostasis Independent of Reproduction Onset

**DOI:** 10.1371/journal.pone.0085964

**Published:** 2014-01-21

**Authors:** Naama Feldman, Libby Kosolapov, Anat Ben-Zvi

**Affiliations:** Department of Life Sciences and The National Institute for Biotechnology in the Negev, Ben-Gurion University of the Negev, Beer Sheva, Israel; Boston University Medical School, United States of America

## Abstract

Protein homeostasis (proteostasis) networks are dynamic throughout the lifespan of an organism. During *Caenorhabditis elegans* adulthood, the maintenance of metastable proteins and the activation of stress responses are inversely associated with germline stem cell proliferation. Here, we employed the thymidylate synthase inhibitor 5-fluoro-2′-deoxyuridine (FUdR) to chemically inhibit reproduction, thus allowing for examination of the interplay between reproduction and somatic proteostasis. We found that treatment with FUdR modulates proteostasis decline both before and after reproduction onset, such that effective induction of the heat shock response was maintained during adulthood and that metastable temperature-sensitive mutant phenotypes were rescued under restrictive conditions. However, FUdR treatment also improved the folding capacity of germline- and gonadogenesis-defective mutants, suggesting that proteostasis modulation by FUdR is independent of germline stem cell proliferation or inhibition of reproduction. Our data, therefore, indicate that FUdR converges on alternative regulatory signals that modulate *C. elegans* proteostasis capacity during development and adulthood.

## Introduction

The long-term health of all cells is dependent on the coordinated activity of protein quality control networks. Despite these protective networks, aging is associated with a general decline in folding capacity and increased susceptibility to protein misfolding diseases. In *Caenorhabditis elegans*, protein homeostasis (proteostasis) is modulated early in adulthood, such that maintenance of metastable proteins and stress-resistance decline sharply following the onset of reproduction [1]–[4].

Reproduction plays a role in determining lifespan, metabolism and proteostasis regulation. Specially, germline stem cells (GSCs) have been suggested to modulate several signaling pathways in the soma, including the *daf-*12 [5]–[9], *daf-16* [10]–[12], *nhr-80* [8], [12], [13], *hsf-1* [14], [15] and TOR [16] pathways. Signals from proliferating GSCs, therefore, coordinate a regulatory network that alters endocrine and metabolic signaling and affects fatty acid metabolism, protein folding, proteasome and autophagy function and lifespan [17]. Indeed, inhibition of GSC proliferation mitigated the functional decline of quality control machineries early in adulthood and resulted in a more effective activation of stress responses and productive maintenance of the cellular proteome during adulthood [14], [16], [18].

To uncover the roles of GSCs in modulating somatic functions, both laser ablation and mutations were previously employed to specifically remove GSCs or inhibit GSC proliferation, respectively [5], [19]. However, as these techniques are both labor-intensive when used for analyzing different genetic backgrounds, a simpler approach was sought to inhibit reproduction and then examine the subsequent impact of such treatment on proteostasis. Given that the thymidylate synthase inhibitor 5-fluoro-2′-deoxyuridine (FUdR) [20] prevents DNA replication and reproduction in *C. elegans* [21] and was shown to prevent over-proliferation of GSCs in adulthood [22], we asked whether FUdR could also affect the induction of a protective heat shock response and protect metastable proteins in adulthood.

FUdR has been customarily used to inhibit reproduction in experiments, such as lifespan assays in which minimal animal handling is desired [21], [23], [24]. However, recent studies have shown that FUdR significantly increases the lifespan of mutant animals, such as *tub-1* and *gas-1* [25], [26], and affects *C. elegans* metabolism [27]. This suggests that FUdR can modulate different signaling pathways that play a role in determining lifespan, possibly in association with the effect of this compound on fertility, even though the lifespan of wild type individuals is not modulated by treatment with this reagent [24]. Here, we show that FUdR treatment not only modulates heat shock response activation and protein folding capacity after reproduction onset but also improves the functions of these protective pathways during development, leading to a rescue of metastable temperature-sensitive mutant phenotypes under restrictive conditions. However, modulation of these proteostasis pathways by FUdR before and after reproduction onset can be dissociated from GSC proliferation or reproduction since FUdR improved these proteostasis functions in germline- and gonadogenesis-defective mutants, suggesting that other signaling pathways can modulate proteostasis during the lifespan of *C. elegans*.

## Results

### FUdR Negates the Sharp Decline in Thermo-resistance that Occurs Early in Adulthood

**Figure 1:**
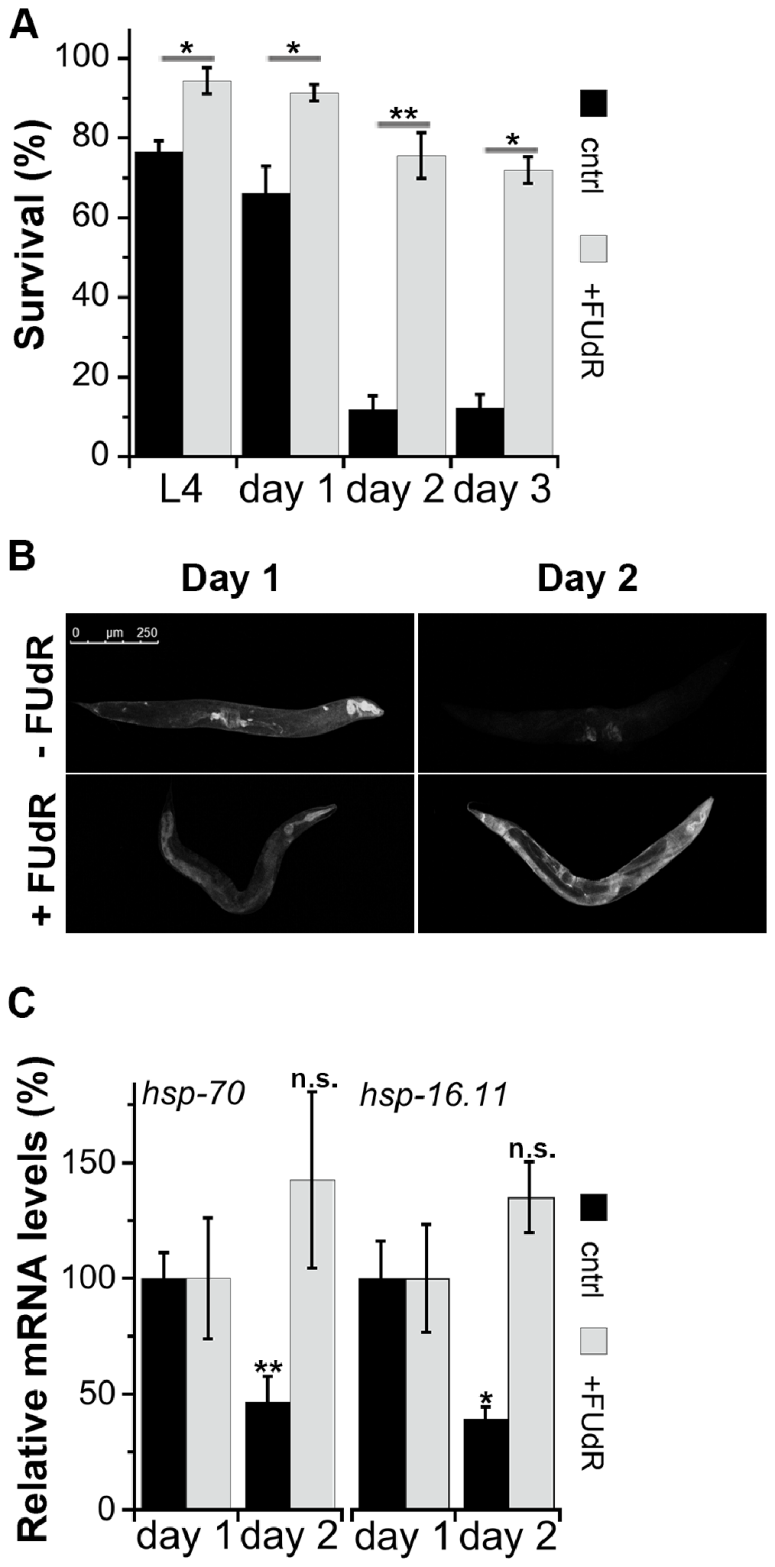
Animals raised on FUdR maintain the ability to mount a protective stress response during adulthood. (A) Age-synchronized wild type (wt) animals raised in the absence (black) or presence (gray) of FUdR were exposed to a 37°C HS for 6 h and survival was assayed. Data represent means ± SEM of >4 independent experiments. *P* values compare age-matched treated and untreated animals. (*) *P*<0.05 and (**) *P*<0.01. (B) Images of age-synchronized wild type animals expressing GFP under control of the *hsp-16.2* promoter (*phsp-16.2::GFP*) raised in the absence or presence of FUdR and subjected to a short HS (90 min at 37°C) on the first or second day of adulthood. Scale bar is 250 µm. (C) Quantification of *hsp-70* (left) and *hsp-16.11* (right) mRNA levels from age-synchronized wild type animals raised in the absence (black) or presence (gray) of FUdR and challenged with a short HS (90 min at 37°C) on the first or second day of adulthood. The data presented are normalized to day 1 of adulthood HS treated animals. Data represent means ± SEM of >3 independent biological samples. *P* values compare mRNA levels on the second day of adulthood with same-treated animals on day 1 of adulthood. (*) *P*<0.05 and (**) *P*<0.01.

Thermo-resistance declines sharply with the onset of *C. elegans* reproduction [1], [14]. The survival rate of wild type nematodes challenged with a prolonged heat shock (HS) on the second day of adulthood was significantly lower than the survival rates of animals at the fourth larval (L4) stage or the first day of adulthood (11.08±3.2% compared to 76.7±2.6% and 66±6.8%, respectively, p<0.005). To test whether FUdR can modify this decline in thermo-resistance after reproduction onset, HS survival rates of wild type animals that had been moved to Nematode Growth Media (NGM) plates supplemented with 100 µg/ml FUdR at the L3 stage (34 h at 25°C) were monitored. This treatment was designed to inhibit reproduction while avoiding the induction of developmental phenotypes associated with earlier exposure to FUdR [21], [23], [24]. When naïve wild type animals raised on FUdR were challenged with prolonged HS on the second or third day of adulthood, survival rates remained high, namely 75.8±5.7% and 71.9±3.3%, respectively (Fig 1A). Even by day 9 of adulthood, 71±5.7% of the FUdR-treated animals were able to survive this stress (data not shown). Such a shift in survival rates was not observed when animals were raised on non-proliferating bacteria, such as ampicillin-sensitive bacteria grown on ampicillin-supplemented plates [28] (Fig. S1). This suggests that FUdR-dependent changes in HS survival are not due to inhibition of bacteria growth but rather result from FUdR-mediated effects on *C. elegans* stress response activation.

To determine whether the increased stress survival observed in FUdR-treated animals was associated with changes in the ability to activate the HS response, we compared the induction of HS genes using a HS transcriptional reporter in which green fluorescent protein (GFP) expression is regulated by the *hsp-16.2* promoter in a stress-dependent manner and then followed GFP expression patterns in wild type animals raised on regular or FUdR-supplemented plates. To complement this approach, mRNA levels of different HS genes were monitored using real-time PCR. When challenged on the first day of adulthood, GFP fluorescence was detected in various somatic tissues but was predominantly observed in intestinal cells of animals raised in the presence or absence of FUdR. FUdR-treated animals maintained their ability to induce *hsp-16.2*-dependent GFP expression, whereas GFP fluorescence was not detected in non-treated animals (Fig. 1B and Fig. S2A). In agreement, the ability of wild type animals raised on regular plates to induce mRNA levels of *hsp-70* and *hsp-16.11* declined strongly on the second day of adulthood, while FUdR-treated animals maintained their ability to induce *hsp-70* and *hsp-16.11* (Fig. 1C). No significant changes were detected in the basal expression levels of HS genes in FUdR-treated animals (Fig. S2B). Likewise, induction of other stress responses, such as oxidative stress response genes (*gcs-1, gst-4*, and *sod-3*), UPR^ER^ (*hsp-4*) and UPR^mt^ (*hsp-6*), was not detected in FUdR-treated animals, suggesting that FUdR-mediated effects on HS response activation are not due to hormesis. These data indicate that treatment with FUdR can regulate the HS response during adulthood but does not elicit the main protein damage responses.

### FUdR Treatment Improved Protein Folding before and after the Onset of Reproduction

FUdR-mediated modulation of thermo-resistance before reproduction onset (L4 and day 1 of adulthood) prompted us to probe the capacity of cellular protein folding in FUdR-treated animals, employing metastable proteins as folding sensors [1]. While proteostasis perturbations induce the misfolding of metastable proteins under permissive conditions, small molecules or genetic modulation that improve proteostasis can rescue metastable protein folding under restrictive conditions [1], [29]–[31]. We, therefore, examined whether treatment with FUdR could rescue the phenotypes of metastable proteins under conditions that induce misfolding.

**Figure 2:**
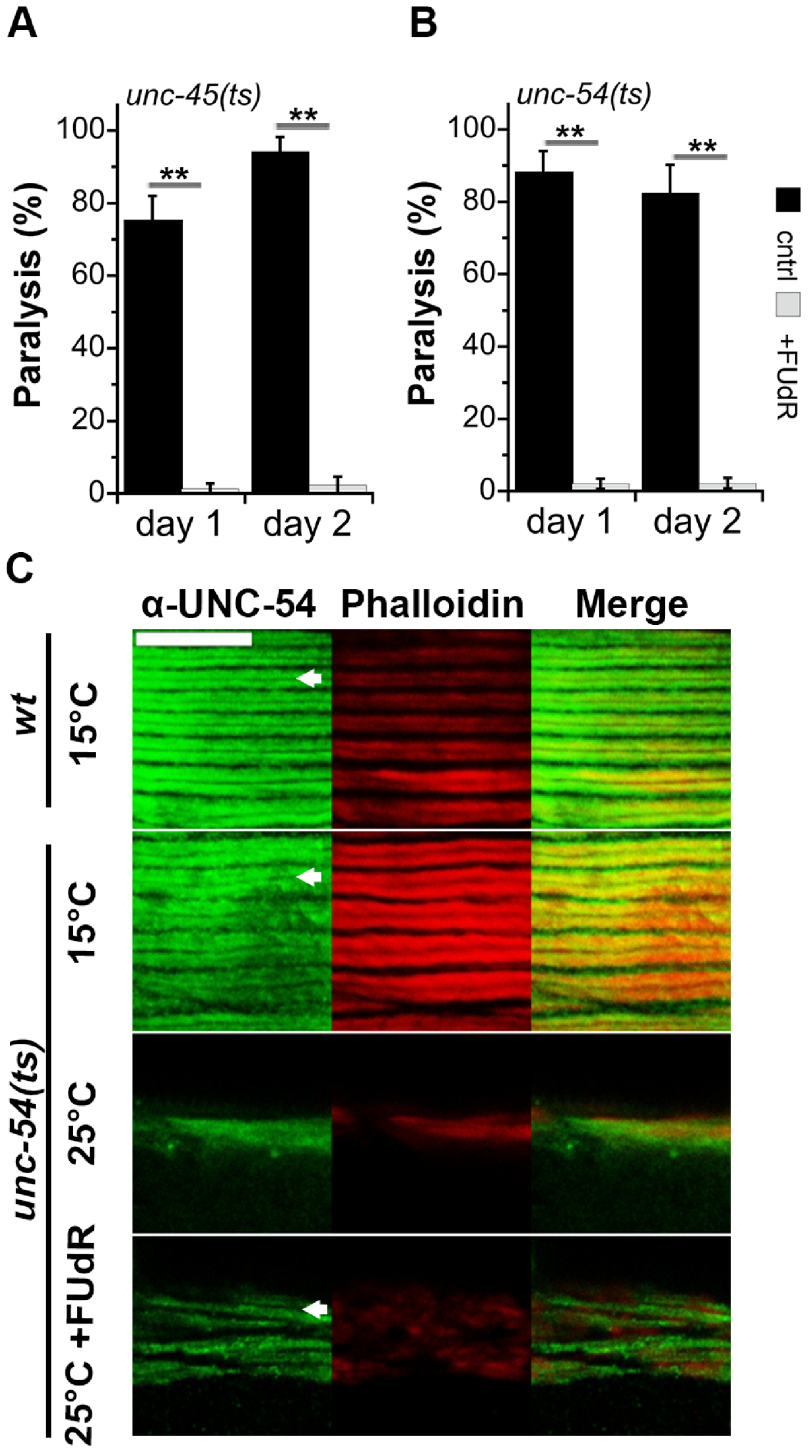
FUdR treatment improved proteostasis before the onset of reproduction. (A–B) The motility of age-synchronized temperature-sensitive *unc-45(e286)* (A), or *unc-54(e1301)* (B) animals raised in the absence (black) or presence (gray) of FUdR was examined on the first and second days of adulthood and the percent of paralyzed animals was scored. Data represent means ± SEM of 5 independent experiments. *P* values compare age-matched treated and untreated animals. (**) *P*<0.01. (C) Confocal images of age-synchronized *unc-54(e1301)* animals raised in the absence or presence of FUdR, and stained with anti-UNC-54 antibodies (green) and Phalloidin (red). Arrows indicate myofilaments. Scale bar is 10 µm.

We first examined the effects of FUdR on metastable temperature-sensitive (ts) mutant proteins expressed during larval development. We employed two metastable missense mutations that result in myosin mislocalization in the sarcomere under restrictive temperatures, namely a mutation in the myosin chaperone *unc-45(e286)* (*unc-45(ts)*) and a mutation in myosin B, *unc-54(e1301)* (*unc-54(ts)*). These temperature-sensitive mutations lead to embryonic lethality and movement defects, the extent of which depends on the developmental stage at which the animals are shifted to the restrictive temperature [32]. To examine how FUdR treatment affected proteostasis during development, embryos were allowed to hatch at 15°C (24 h) and were then shifted to 25°C (L1–L2 larvae) to avoid embryo lethality. These animals were subsequently moved to plates containing FUdR after 24 h at 25°C (L3 larvae) or maintained on regular NGM plates. Motility was examined on the first and second days of adulthood. As expected, 75.5±6.5% and 88.3±5.7% of the *unc-45(ts)* and *unc-54(ts)* animals, respectively, that were grown under restrictive conditions (25°C) in the absence of FUdR were paralyzed by the first day of adulthood. In contrast, treatment with FUdR resulted in a complete rescue of paralysis of both *unc-45(ts)* and *unc-54(ts)* animals, with 1.4±1.4% and 2.1±1.4% of the population being paralyzed, respectively. At the same time, the mobility of the wild type animals was unaffected (data not shown). Motility was also maintained on the second day of adulthood, with 2.3±2.3% and 2.2±1.5% of these populations being paralyzed, respectively (Fig. 2A and 2B).

The motility decline of *unc-54(ts)* mutant animals is associated with a severe disruption of myofilament organization [1]. We, therefore, performed immunostaining with UNC-54-specific antibodies (28-2) to monitor myosin localization in animals raised under restrictive conditions in the presence or absence of FUdR. As expected, the myofilaments of *unc-54(ts)* animals shifted to 25°C were disrupted and UNC-54 was completely mislocalized, compared to wild type or *unc-54(ts)* animals raised at 15°C. In contrast, myofilaments of FUdR-treated animals were partially maintained, and showed reduced number of myo-filaments (Fig. 2C). These observations support our finding that FUdR treatment modulated proteostasis during development, thereby increasing both stress resistance and folding capacity.

**Figure 3:**
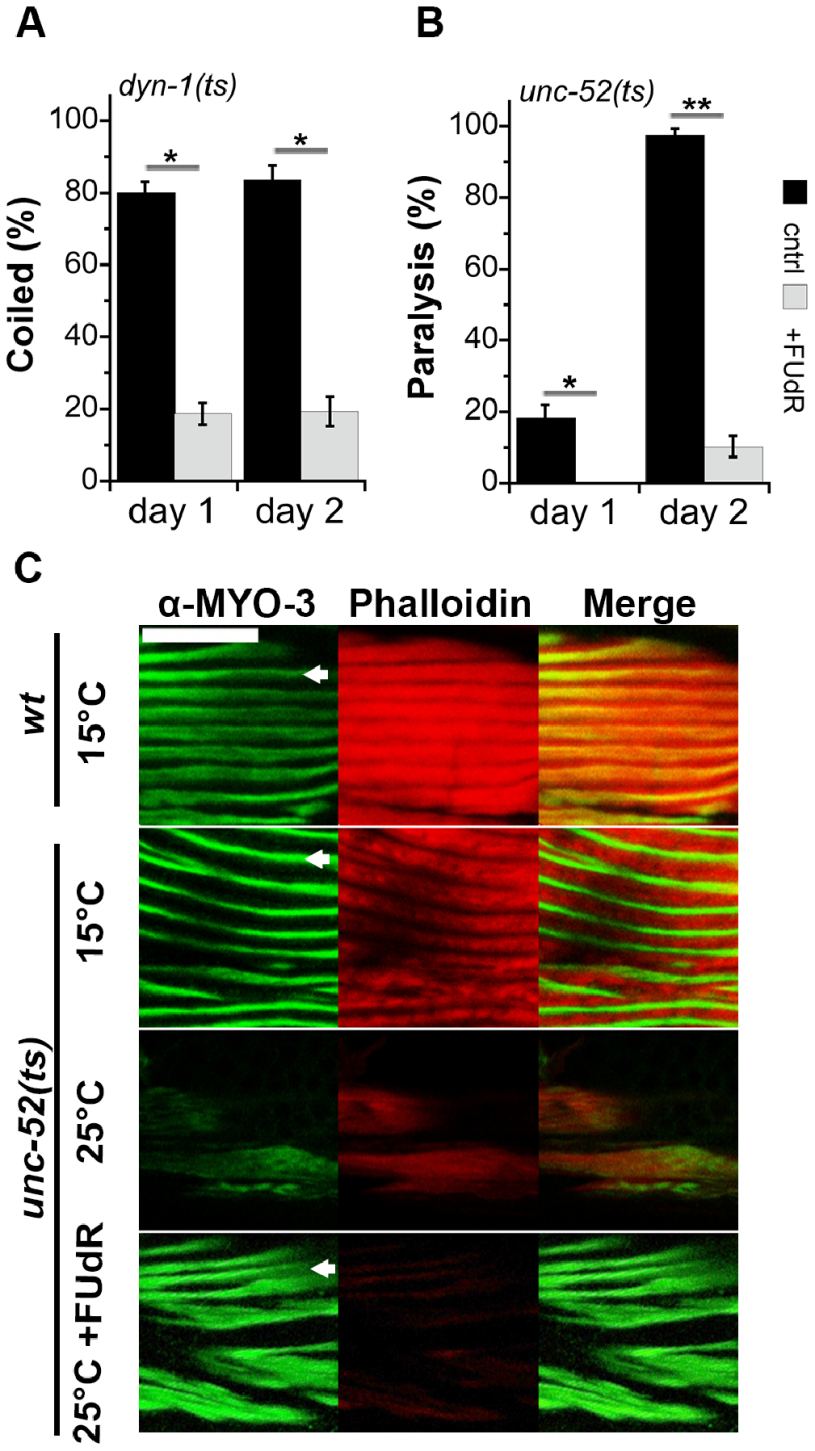
FUdR treatment improved proteostasis after the onset of reproduction. (A) Age-synchronized *dyn-1(ky51)* animals raised in the absence (black) or presence (gray) of FUdR were shifted to 28°C on the first or second days of adulthood and the percent of coiled animals was scored. Data represent means ± SEM of 4 independent experiments. *P* values compare age-matched treated and untreated animals. (*) *P*<0.05. (B) The motility of age-synchronized temperature-sensitive *unc-52(e669su250)* animals raised in the absence (black) or presence (gray) of FUdR was examined on the first and second days of adulthood and the percent of paralyzed animals was scored. Data represent means ± SEM of 5 independent experiments. *P* values compare age-matched treated and untreated animals. (*) *P*<0.05 and (**) *P*<0.01. (C) Confocal images of age-synchronized *unc-52(e669su250)* animals raised in the absence or presence of FUdR, and stained with anti-MYO-3 antibodies (green) and phalloidin (red). Arrows indicate myofilaments. Scale bar is 10 µm.

We then examined the effects of FUdR treatment on protein folding after the onset of reproduction by monitoring a temperature-sensitive mutation in dynamin-1 *dyn-1(ky51)*, a protein that functions in endocytosis and in neuronal synaptic vesicle recycling. This mutation leads to a rapid and reversible arrest of endocytosis when animals are shifted to 25°C [1], [33]. 83.7±3.9% of *dyn-1(ts)* mutant animals that were raised at 20°C coiled around themselves when shifted to 28°C on day 2 adulthood. In contrast, animals treated with FUdR were mostly unaffected, with only 19.3±4% of *dyn-1(ts)* mutant animals showing coiling in response to this shift (Fig. 3A).

Similar behavior was observed for animals presenting a temperature-sensitive mutation in perlecan, *unc-52(e669su250)*. This mutation leads to detachment of myo-filamants from the basal membrane in body wall muscle of adult *C. elegans* and results in paralysis under restrictive conditions (growth at 25°C) [1]. This mutation is in a splicing variant that is only expressed in adulthood. When animals expressing *unc-52(ts)* are raised at 25°C, they become paralyzed starting from day 1 of adulthood, allowing us to differentiate the effects of FUdR before and after the onset of reproduction. The motility of mutant animals expressing *unc-52(ts)* declined quickly, such that these animals were completely paralyzed (97.7±1.6%) and showed reduced number of myo-filaments by day 2 of adulthood when maintained under restrictive conditions. In contrast, only 10.3±4.4% of animals grown on FUdR were paralyzed, with myo-filament structure being maintained in such individuals, similar to wild type animals (Fig. 3B and 3C). Thus, FUdR treatment can rescue the folding of metastable proteins before and after the onset of reproduction.

### FUdR-mediated Effects on Thermo-resistance and Protein Folding are Independent of the GSC Pathway

**Figure 4:**
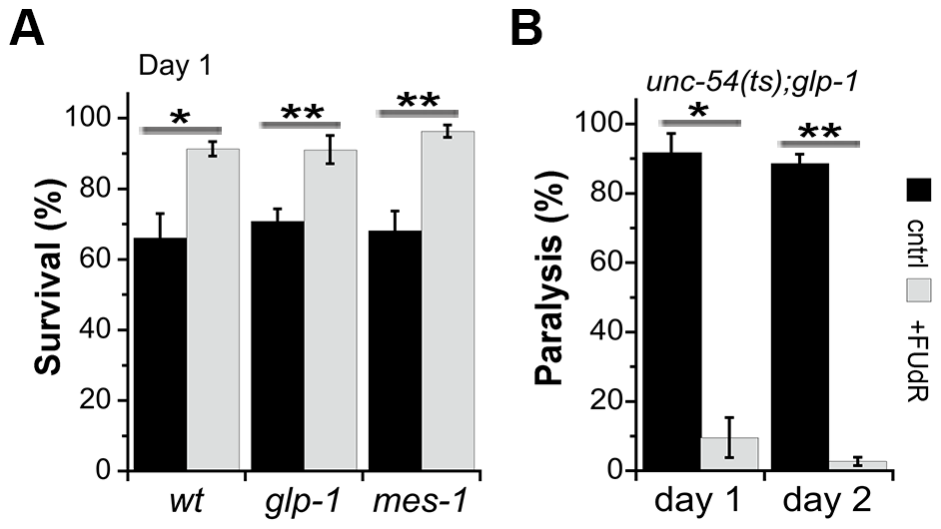
FUdR effects on proteostasis during development are independent of GSC inhibition. (A) Age-synchronized *glp-1(e2141)* animals raised in the absence (black) or presence (gray) of FUdR were exposed to a 37°C HS for 6 h on the first day of adulthood and survival was assayed. Data represent means ± SEM of >5 independent experiments. *P* values compare age-matched treated and untreated animals. (*) *P*<0.05 and (**) *P*<0.01. (B) The motility of age-synchronized *unc-54(e1301);glp-1(e2141)* animals raised in the absence (black) or presence (gray) of FUdR was examined on the first and second days of adulthood and the percent of paralyzed animals was scored. Data represent means ± SEM of >3 independent experiments. *P* values compare age-matched treated and untreated animals. (*) *P*<0.05 and (**) *P*<0.01.

HS survival rates of FUdR-treated animals on day 2 of adulthood were similar to those observed previously for GSC proliferation mutant *glp-1(e2141ts*) (*glp-1*) sterile animals [14]. However, unlike the *glp-1* animals, the survival rates of FUdR-treated animals were also improved at L4 and day 1 of adulthood, where 94.4±3.3% and 91.4±2.1% of the animals survived, respectively, as compared to untreated animals (p<0.05) (Fig. 1A). Given that *glp-1* animals did not show any effects on thermo-resistance before the onset of reproduction [14] whereas FUdR-treated animals did, we examined the effects of FUdR treatment on the HS survival of *glp-1* animals before the onset of reproduction. If FUdR functions by inhibiting GSC proliferation, then FUdR treatment would not be expected to affect *glp-1* mutant animals. In contrast, were FUdR to function in a parallel pathway to that used for GSC inhibition, then *glp-1* animals treated with FUdR should display an additive effect on thermo-tolerance upon treatment with this reagent. Treatment of *glp-1* animals with FUdR resulted in a significant increase in HS survival on day 1 of adulthood, with 91.1±4% of the *glp-1* animals treated with FUdR surviving, as compared to 70.9±3.5% of the non-treated animals (p<0.01) (Fig. 4A). Increased thermo-resistance was also observed when *mes-1(bn7) (mes-1)* individuals, corresponding to a different germline proliferation mutant, were treated with FUdR (96.1±2%, as compared to 68.1±5.6% for untreated animals, p<0.005) (Fig. 4A). This increase in survival was similar to that observed following FUdR treatment of wild type animals (Fig. 4A), suggesting that FUdR-mediated effects on proteostasis before the onset of reproduction occur independently of GSC inhibition. In agreement with this proposal, it was noted that when *unc-54(ts)* animals were crossed with *glp-1* mutant animals, 91.8±2% of the *unc-54(ts);glp-1* mutant progeny were paralyzed on the first day of adulthood, with FUdR treatment completely rescuing the paralysis phenotype (2.7.±1.2% on day 2 of adulthood, p<0.005) of these animals (Fig. 4B).

**Figure 5:**
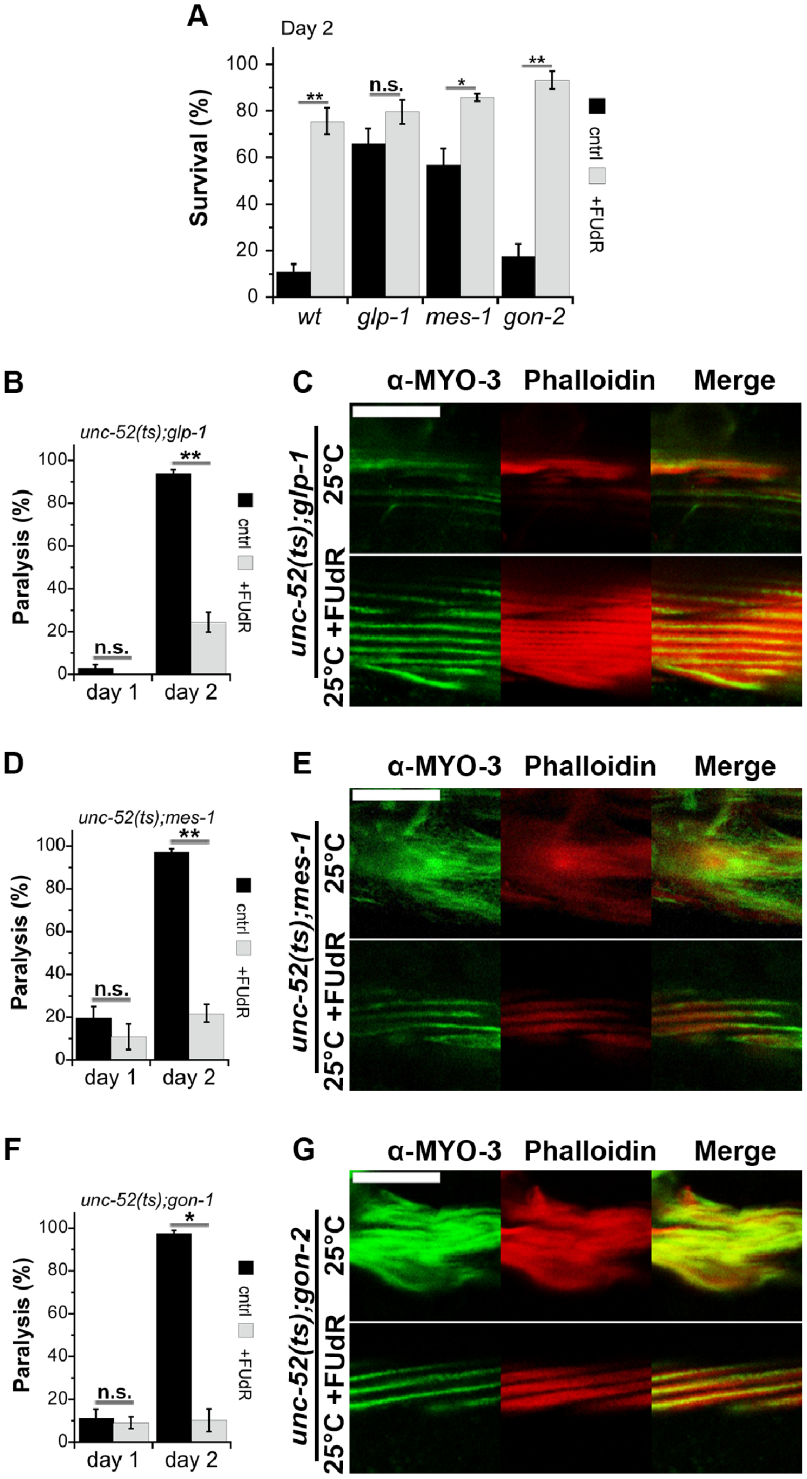
FUdR treatment modulates proteostasis of germline- and gonadogenesis-defective mutants upon transition to adulthood. (A) Age-synchronized *glp-1(e2141)*, *mes-1(bn7)* or *gon-2(q388)* animals raised in the absence (black) or presence (gray) of FUdR were exposed to a 37°C HS for 6 h on the second day of adulthood and survival was assayed. Data represent means ± SEM of >5 independent experiments. *P* values compare age-matched treated and untreated animals. (*) *P*<0.05, (**) *P*<0.01 and (n.s.) not significant. (B) The motility of age-synchronized *unc-52;glp-1* animals raised in the absence (black) or presence (gray) of FUdR was examined on the second day of adulthood and the percent of paralyzed animals was scored. Data represent means ± SEM of >4 independent experiments. *P* values compare age-matched treated and untreated animals. (*) *P*<0.05, (**) *P*<0.01 and (n.s.) not significant. (C) Confocal images of age-synchronized *unc-52;glp-1* animals raised in the absence or presence of FUdR, and stained with anti-MYO-3 antibodies (green) and Phalloidin (red). Scale bar is 10 µm. (D) The motility of age-synchronized *unc-52;mes-1* was examined as in B. (E) Confocal images of age-synchronized *unc-52;mes-1* as in C. (F) The motility of age-synchronized *unc-52;gon-2* was examined as in B. (G) Confocal images of age-synchronized *unc-52;gon-2* as in C. Scale bar is 10 µm.

We then examined the effects of FUdR treatment on *glp-1* animals after the onset of reproduction. FUdR-treated *glp-1* animals showed a small increase in survival rates (77.8±4%, as compared to 66±6.5% for non-treated animals) when examined on day 2 of adulthood (Fig. 5A). Prolonging the HS to 9 hours increased this difference, with 57.2±4% survival being noted following treatment, as compared to 35.9±6.4% (p<0.005) for *glp-1* non-treated animals (Fig. S3). Likewise, *mes-1* animals treated with FUdR showed increased thermo-resistance in the face of prolonged HS (87.5±2%, compared to 62.9±7.9% for untreated animals, p<0.05), suggesting that the thermo-resistance improvement observed in FUdR-treated animals occurs independently of GSC inhibition (Fig. 5A).

To extend this analysis, *unc-52(ts)* animals were crossed with GSC mutant *glp-1-* or *mes-1*-expressing animals and paralysis was monitored on day 2 of adulthood. 94.2±1.4% of the *unc-52(ts);glp-1* mutant animals and 97.2±1.4% of the *unc-52(ts);mes-1* mutant animals were paralyzed when cultivated at 25°C, whereas the paralysis phenotype of *unc-52(ts);glp-1* or *unc-52(ts);mes-1* animals treated with FUdR was reduced to 18.4±2.4% (p<0.005) and 23±4.5% (p<0.05), respectively. Moreover, myo-filament organization was partially maintained even though the animals were grown under restrictive conditions (Fig. 5B–5E). Thus, FUdR treatment rescued the proteostasis decline that occurred in adulthood, independent from inhibition of GSC proliferation.

### FUdR Treatment can Improve Thermo-resistance and Protein Folding of Gonadogenesis-defective Mutants

FUdR inhibits reproduction [23]. Animals exposed to FUdR using our treatment protocol (i.e. a shift to FUdR-containing surroundings after 34 h at 25°C) showed some germline stem cell proliferation but no progeny production (Fig. S4), suggesting that other stages of reproduction might be affected by FUdR. Given that removal of the gonad also affects some aspects of somatic function [34], we reasoned that FUdR might affect proteostasis by inhibiting reproduction in a GSC-independent manner. We, therefore, examined the HS survival of gonadogenesis-defective mutants *gon-2(q388)* (*gon-2*) and asked whether proteostasis in animals that lack a reproductive system was modulated by FUdR.

Similar to wild type and *glp-1* animals, the survival rates of *gon-2* animals treated with FUdR were elevated on day 1 of adulthood (84.9±7.5%, as compared to 65±7% for untreated animals, p<0.005). Likewise, the survival rates of *gon-2* animals that declined on day 2 of adulthood (9.7±1.8%, a value similar to what is seen with wild type) were rescued by FUdR treatment (62.6±7.7%) (Fig. 5A). FUdR treatment also rescued the paralysis of *unc-52(ts);gon-2* animals from 97.5±1.3% to 10.3±4.8% (p<0.05). In agreement, myo-filament organization in FUdR-treated animals was partially preserved even under restrictive conditions (Fig. 5F and 5G). Thus, FUdR treatment improved protein-folding capacity in adulthood in the absence of a reproductive system, suggesting that FUdR-mediated effects on proteostasis are not necessarily related to its effects on reproduction. Indeed, a significant improvement in thermo-resistance was observed even when FUdR concentrations were reduced 10-fold to a concentration that does not inhibit reproduction (Fig. S5) [21], [25]. We, therefore, propose that other signaling pathways, independent of reproduction, exist and that these can modulate proteostasis capacity during adulthood, as well as during development.

## Discussion

Here, we showed that FUdR is a potent modulator of proteostasis in *C. elegans*. Treatment with FUdR increased HS response activation both before and after the onset of *C. elegans* reproduction. This was not associated with an induction of protein damage responses, such as the HSR or UPR, and resulted in a maintenance of the folding and function of temperature-sensitive mutant proteins under restrictive conditions. Moreover, we showed that FUdR-mediated actions during development and adulthood occur independently from the effects of this compound on reproduction. While GSC proliferation mutants modulated proteostasis only during adulthood [14], [16], [18], FUdR treatment improved proteostasis of wild type as well as germline- and gonadogenesis-defective mutant animals both before and after the onset of reproduction. These data suggest that FUdR affects proteostasis via an alternative pathway unrelated to GSC or gonadal signaling. In agreement, FUdR was found to increase the lifespan of *glp-1* animals [25]. Given that nutrient availability and environmental challenges were also shown to reset stress response activation and modulate the capacity of the cellular maintenance machinery [34]–[38], we propose that a switch in quality control function can result from a combination of exogenous and endogenous signals, possibly acting at specific times point during development [39] or adulthood [1], [4], [40], that dictate the ability of the organism to maintain its proteome.

How then does FUdR modulate proteostasis? FUdR inhibits thymidylate synthase, [20], resulting in an imbalance of deoxyribonucleoside triphosphate pools and causing accumulation of DNA double strand breaks [41]. Recent publications have shown that induction of the DNA damage response in *C. elegans* germline stem cells transpires in direct response to DNA damage by inactivating checkpoint proteins, blocking programmed cell death or upon impairing nucleotide excision repair and that such damage repair leads to improved HS survival [35], [36], [42], [43]. Signals to the soma are suggested to function via the induction of an innate immune response [35], while somatic signals acting via KRI-1 are thought to regulate germline stem cell apoptosis [42]. This in turn suggests that DNA damage responses in the germline can be linked to improved somatic proteostasis. However, we find that germline stem cells are not required for FUdR to confer somatic resistance. Indeed, FUdR treatment was able to enhance stress survival and proteostasis even in *gon-2* animals that lack the entire reproductive system to a similar extent as seen in intact animals. Thus, FUdR is not likely to affect HS activation by inducing DNA damage responses in the germline. Given that ionizing radiation caused DNA damage to somatic cells of wild type and *glp-1* animals but did not activate the DNA damage response and induce programmed cell death [44], we cannot exclude the possibility that FUdR-dependent DNA damage in somatic cells modulates thermo-resistance and protein folding in the soma.

Treatment with amyloid-binding compounds, such as Thioflavin T (ThT), also enhanced cellular proteostasis during *C. elegans* development and adulthood. Indeed, ThT treatment could rescue the misfolding of *unc-52(ts)* and *unc-54(ts)* mutant animals grown under restrictive conditions, both before and after the onset of reproduction, similarly to what was observed here upon FUdR treatment [29]. ThT was suggested to induce stress-response signaling directly by acting as a “stress response mimetic”. Likewise, NG-094, a hydroxylamine derivative, was shown to potentiate HSF-1 function and improve proteostasis [45]. Thus, FUdR may modulate different modes of cellular stress signaling and improve organismal stress survival and proteostasis capacity. Indeed, a metabolomics analysis of FUdR-treated animals showed that FUdR treatment had a significant effect on the profile of metabolites [27]. Given that changes in lipid composition can modulate proteostasis [16], [46], it is possible that FUdR modulates different quality control systems by modulating cellular metabolism. Additional studies are required to determine which pathway or pathways modulate proteostasis in a FUdR-dependent manner. However, our data clearly demonstrate that FUdR, even at low concentrations, changes the ability of *C. elegans* to respond to the environment and, therefore, suggest the use of caution when employing FUdR to inhibit reproduction and maintain a synchronized population [25]–[27].

## Materials and Methods

### Nematodes and Growth Conditions

For a list of strains used in this work and name abbreviations, see Table S1. Nematodes were grown on NGM plates seeded with *Escherichia coli* OP50-1 strains at 15°C. Unless otherwise stated, embryos laid at 15°C were set on new plates at 25°C. After 34 h, the animals were transferred to control or 100 µg/ml FUdR-containing plates, unless stated otherwise. The first day of adulthood (day 1) was set at 50 h after temperature shift, before the onset of egg laying. Animals were moved every one or two days during the reproductive period to avoid progeny contamination.

### Statistical Analysis

Experiments were repeated at least 3 times and data are presented as means ± SEM. *P* values were calculated by using the Wilcoxon Mann-Whitney rank sum test to compare two independent populations. Data were compared to age-matched untreated animals. For Figure 1C, data were compared to those obtained with same-treated animals on day 1 of adulthood. (*) denotes *P*<0.05 and (**) denotes *P*<0.01.

### HS Treatment

A total of 15–40 age-synchronized animals grown at 25°C were used for each assay. Animals were moved to new plates, which were then sealed and placed in a 37°C bath for 90 min. Animals were frozen or fixed immediately following stress.

### Thermo-resistance Assay

Animals were picked at the indicated age and transferred to a 24-well plate containing HS buffer (100 mM Tris-HCl, pH 7.4, 17 mM NaCl and 1% cholesterol supplemented with bacteria). Animals were then subjected to a 37°C HS for 6 h (or 9 h in the experiment depicted in Fig. S3). HS buffer was supplemented with SYTOX orange (Invitrogene) and animal survival was scored by monitoring dye uptake using a Leica M165 FC fluorescent stereoscope with a TXR filter. Fluorescent animals were scored as dead. >45 animals per experimental condition were scored.

### RNA Levels

Total RNA was extracted from wild type animals raised in the absence or presence of FUdR that were untreated or subjected to HS (see HS treatment). RNA was extracted using the TRIzol reagent (Invitrogene). For cDNA synthesis, mRNA was reverse transcribed using the iScriptTM cDNA Synthesis Kit (Bio-Rad). Quantitative PCR was performed on a C1000 Thermal Cycler (Bio-Rad) with SsoFas EvaGreen Supermix (Bio-Rad).

### GFP Stress Reporters

Animals expressing GFP under control of the *hsp-16.2* promoter (*phsp-16.2::GFP*) [47] were subjected to HS (see HS treatment) and GFP fluorescence in the gut was monitored 18–24 h later using a Leica M165 FC fluorescent stereoscope with a GFP2 filter and capture using Leica DFC360FX fluorescent camera. Likewise, GFP fluorescence in the gut of untreated wild type animals raised in the absence or presence of FUdR was examined.

### Paralysis Assay

A total of 15–40 age-synchronized animals were used for each assay. Animals were moved to clean plates containing FUdR or not every day and paralysis was scored by monitoring animal movement after 10 min. Those animals that did not move were scored as paralyzed. >45 animals were scored per experimental condition.

### Coiling Assay

*dyn-1(ts)* animals were raised at 20°C and 15–40 L3 animals were set on plates in the presence or absence of FUdR. The animals were then shifted to plates equilibrated to 28°C for 5 min and the percentage of animals that were coiled was scored. >50 animals were scored per experimental condition.

### Immunostaining, Phalloidin and DAPi Staining

Immunofluorescence studies were performed as previously described [1]. Animals were stained with 28-2 anti-UNC-54 (a gift from Prof. Barral) or 5–6 anti-MYO-3 antibodies (Hybridoma Bank) [48]. Secondary Dylight 488-conjugated goat anti-mouse IgG antibodies (Jackson Immuno Research) were used to reveal primary antibody binding. Animals were also stained with Rhodamine-Phalloidin to mark actin. Animals were imaged using a Leica DM550 confocal microscope through a 63×1.0 numerical aperture objective with a 488-nm or a 532-nm line for excitation. For DAPI staining (#40043, Biotium), animals were fixed as previously described [1] and were then incubated overnight at 4°C with 20 µg/ml DAPI in x1PBS. Animals were imaged using an Olympus Fluoview FV1000 confocal microscope through a 60×1.0 numerical aperture objective set with a 405 nm line for excitation.

## Supporting Information

Figure S1 **HS Survival rates of wild type animals fed on non-proliferating ampicillin-treated bacteria.** Age-synchronized wild type animals raised on FUdR- or ampicillin-supplemented plates were exposed to a 37°C HS for 6 h and survival was assayed on day 2 of adulthood. Data represent means ± SEM of >4 independent experiments. *P* values compare age-matched treated and untreated animals. (*) *P*<0.05.
(TIF)

Figure S2 **FUdR treatment does not induce stress responses associated with damaged proteins.** (A) Images of age-synchronized wild type animals expressing GFP under control of the *hsp-16.2* promoter (*phsp-16.2::GFP*) raised in the absence or presence of FUdR on the first or second day of adulthood. (B) Quantification of mRNA levels from age-synchronized wild type animals raised in the absence (black) or presence (gray) of FUdR on the first day of adulthood. The data presented are normalized to those obtained with non-treated animals. Data represent means ± SEM of >3 independent biological samples. *P* values compare age-matched treated and untreated animals. No significant difference in expression levels was observed for genes, but for *hsp-4*, which decreased upon FUdR treatment. (*) *P*<0.05.
(TIF)

Figure S3 **FUdR can further improve HS Survival of** ***glp-1*** **animals.** Age-synchronized wild type or *glp-1* animals raised in the absence (black) or presence (gray) of FUdR were exposed to a 37°C HS for 9 h on day 2 of adulthood and survival was assayed. Data represent means ± SEM of >4 independent experiments. *P* values compare age-matched treated and untreated animals. (*) *P*<0.05.
(TIF)

Figure S4 **FUdR affects GSC proliferation. Confocal images of age-synchronized day 2 adults, wild type animals raised in the absence or presence of FUdR and stained with DAPI.**
(TIF)

Figure S5 **The effects of FUdR concentrations on the ability to mount a protective HS response.** (A–B) Age-synchronized wild type animals raised on plates containing different concentrations of FUdR (as indicated) were exposed to a 37°C HS for 6 h on the first (A) or second (B) day of adulthood and survival was assayed. Data represent means ± SEM of >4 independent experiments. *P* values compare age-matched treated and untreated animals. (*) *P*<0.05 and (**) *P*<0.01.
(TIF)

Table S1 Strains used in this work.
(PDF)
