# Supplementary figures and images for: Fluorodeoxyuridine Improves Caenorhabditis elegans Proteostasis Independent of Reproduction Onset
Source: PLoS One. 2014 Jan 21;9(1):e85964. doi: 10.1371/journal.pone.0085964 (PMC3897603; doi:10.1371/journal.pone.0085964)

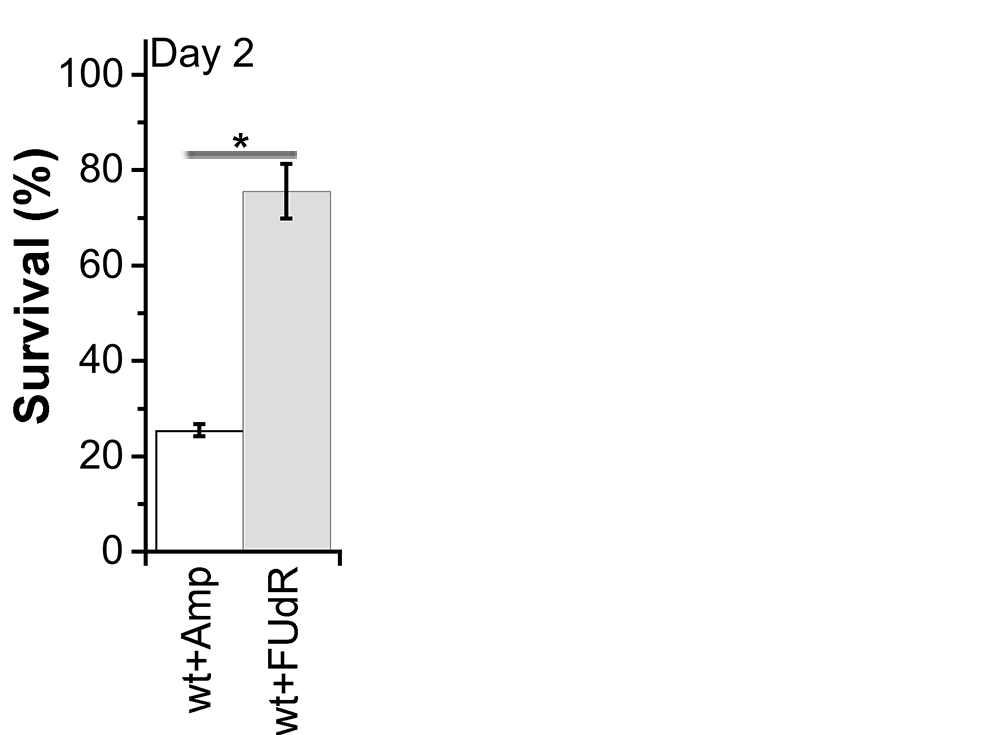

Supplement: Figure S1 — HS Survival rates of wild type animals fed on non-proliferating ampicillin-treated bacteria. Age-synchronized wild type animals raised on FUdR- or ampicillin-supplemented plates were exposed to a 37°C HS for 6 h and survival was assayed on day 2 of adulthood. Data represent means ± SEM of >4 independent experiments. P values compare age-matched treated and untreated animals. (*) P<0.05. (TIF) [file pone.0085964.s001.tif]

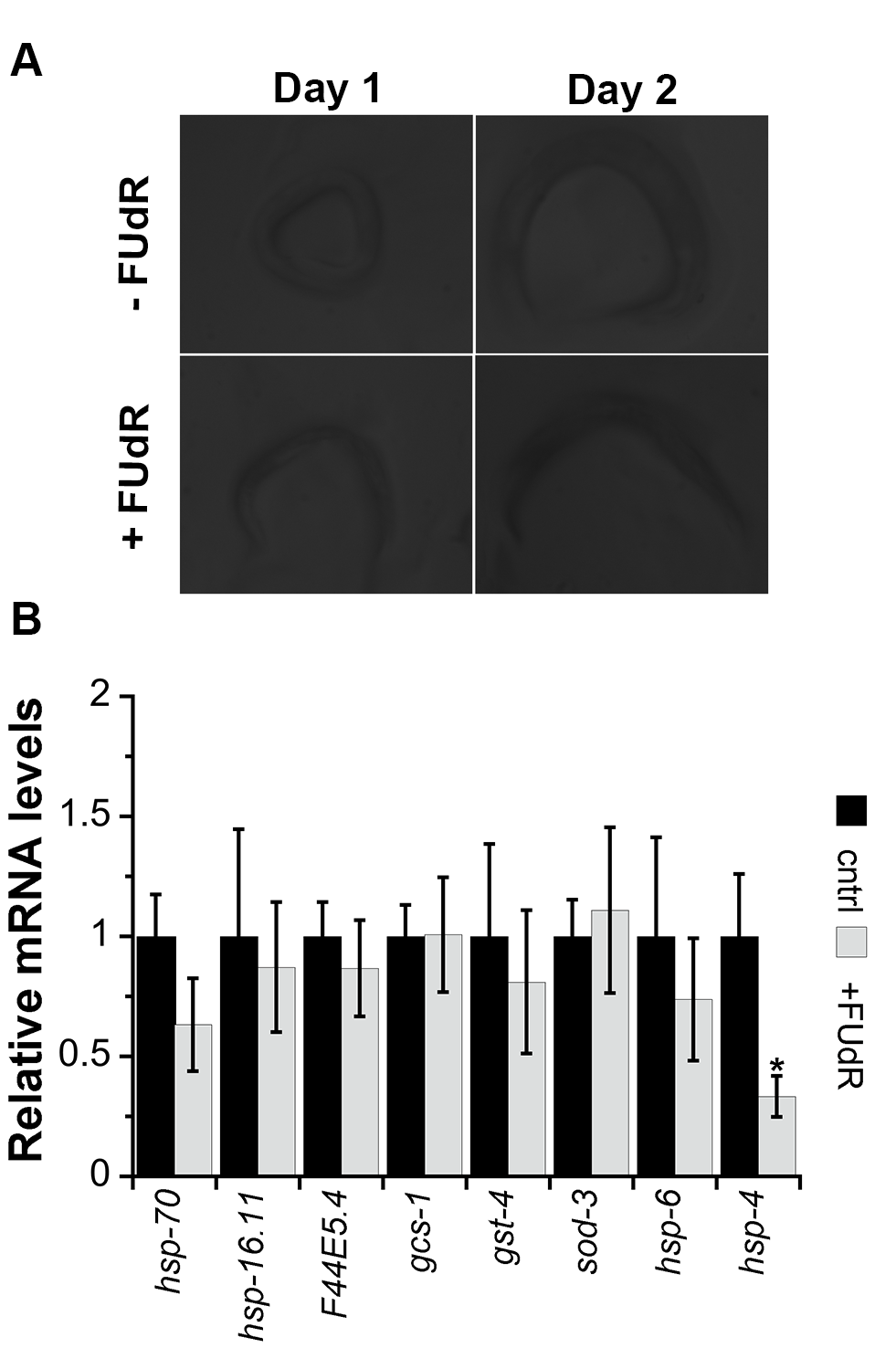

Supplement: Figure S2 — FUdR treatment does not induce stress responses associated with damaged proteins. (A) Images of age-synchronized wild type animals expressing GFP under control of the hsp-16.2 promoter (phsp-16.2::GFP) raised in the absence or presence of FUdR on the first or second day of adulthood. (B) Quantification of mRNA levels from age-synchronized wild type animals raised in the absence (black) or presence (gray) of FUdR on the first day of adulthood. The data presented are normalized to those obtained with non-treated animals. Data represent means ± SEM of >3 independent biological samples. P values compare age-matched treated and untreated animals. No significant difference in expression levels was observed for genes, but for hsp-4, which decreased upon FUdR treatment. (*) P<0.05. (TIF) [file pone.0085964.s002.tif]

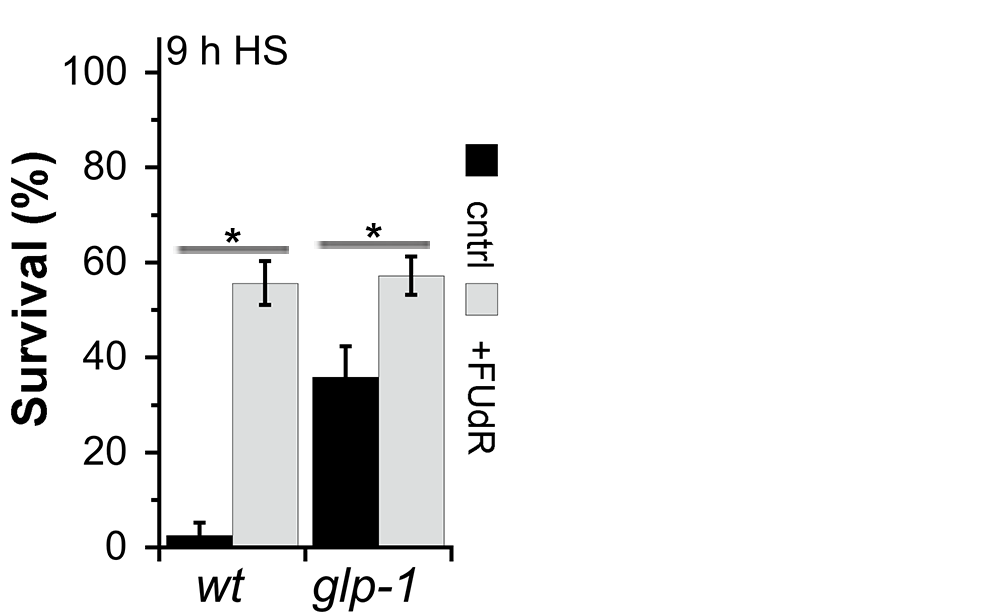

Supplement: Figure S3 — FUdR can further improve HS Survival of glp-1 animals. Age-synchronized wild type or glp-1 animals raised in the absence (black) or presence (gray) of FUdR were exposed to a 37°C HS for 9 h on day 2 of adulthood and survival was assayed. Data represent means ± SEM of >4 independent experiments. P values compare age-matched treated and untreated animals. (*) P<0.05. (TIF) [file pone.0085964.s003.tif]

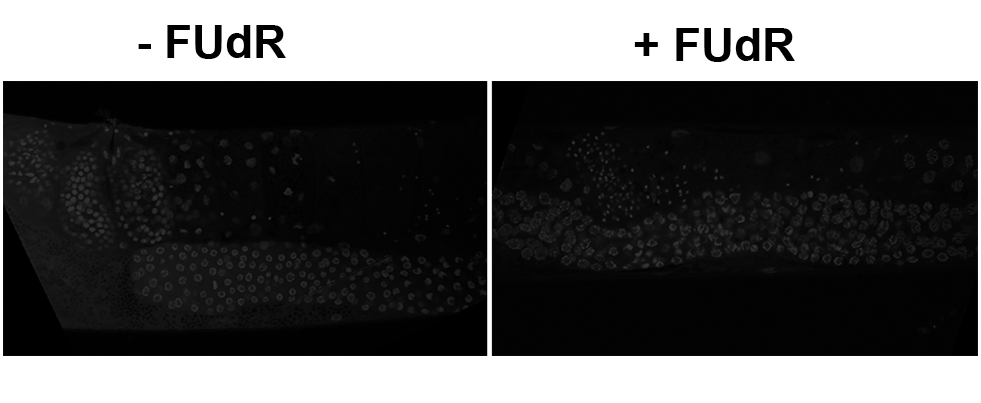

Supplement: Figure S4 — FUdR affects GSC proliferation. Confocal images of age-synchronized day 2 adults, wild type animals raised in the absence or presence of FUdR and stained with DAPI. (TIF) [file pone.0085964.s004.tif]

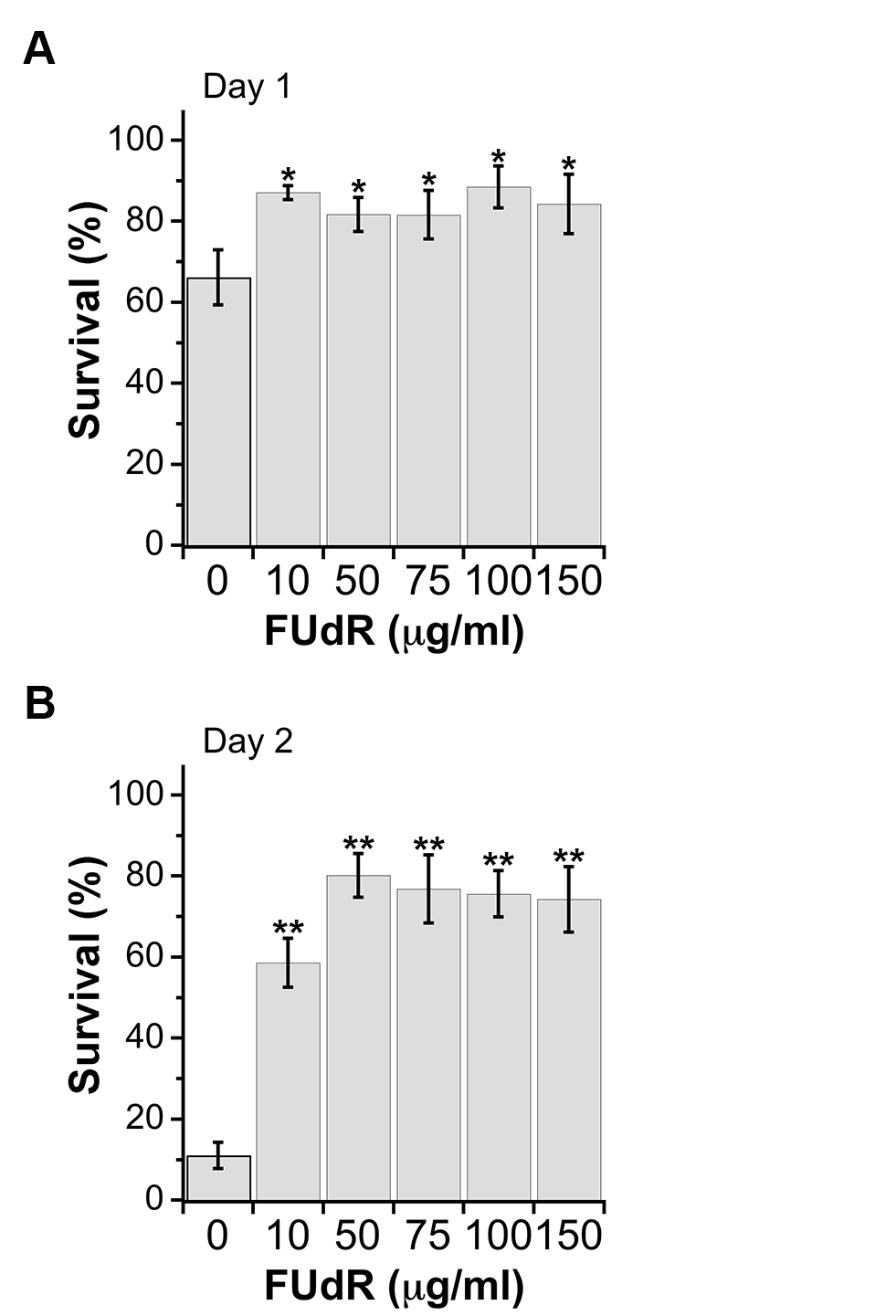

Supplement: Figure S5 — The effects of FUdR concentrations on the ability to mount a protective HS response. (A–B) Age-synchronized wild type animals raised on plates containing different concentrations of FUdR (as indicated) were exposed to a 37°C HS for 6 h on the first (A) or second (B) day of adulthood and survival was assayed. Data represent means ± SEM of >4 independent experiments. P values compare age-matched treated and untreated animals. (*) P<0.05 and (**) P<0.01. (TIF) [file pone.0085964.s005.tif]
