# Supplementary material for: Fluorodeoxyuridine Improves Caenorhabditis elegans Proteostasis Independent of Reproduction Onset
Source: PLoS One. 2014 Jan 21;9(1):e85964. doi: 10.1371/journal.pone.0085964 (PMC3897603; doi:10.1371/journal.pone.0085964)
Supplement: Table S1 — Strains used in this work. (PDF) [file pone.0085964.s006.pdf]

**Table S1. Strains used in this work**			
**Strain**	**Abbreviation**	**Genotype**	**Phenotype**
N2	*wt*	---	---
CF1903	*glp-1*	*glp-1(e2141)*	ts GSC arrest, sterile
SS149	*mes-1*	*mes-1(bn7)*	ts GSC arrest, sterile
CF2253	*gon-2*	*gon-2*(q388ts)	ts gonadless, sterile
CL2070	*phsp-16.2::GFP*	*dvIs70[phsp-16.2::gfp; rol-6(su1006)]*	HS reporter, GFP expression, roller
CB286	*unc-45(ts)*	*unc-45(e286)*	ts slow moving UNC
CB1301	*unc-54(ts)*	*unc-54(e1301)*	ts slow moving UNC
HE250	*unc-52(ts)*	*unc-52(e669su250)*	ts adult onset UNC
ABZ8	*unc-54(ts);glp1*	*unc-54(e1301); glp-1(e2141)*	ts UNC, ts GSC arrest, sterile
ABZ92	*unc-52(ts); glp-1*	*unc-52(e669su250); glp-1(e2141)*	ts UNC, ts GSC arrest, sterile
ABZ94	*unc-52(ts);mes-1*	*unc-52(e669su250); mes-1(bn7)*	ts UNC, ts GSC arrest, sterile
ABZ93	*unc-52(ts); gon-2*	*unc-52(e669su250); gon-2(q388)*	ts adult onset UNC, ts gonadless, sterile

Abbreviations: ts - temperature sensitive, GSC - germline stem cell, HS - heat shock, UNC- uncoordination.
